# Excessive reactive oxygen species are therapeutic targets for intervertebral disc degeneration

**DOI:** 10.1186/s13075-015-0834-8

**Published:** 2015-11-05

**Authors:** Satoshi Suzuki, Nobuyuki Fujita, Naobumi Hosogane, Kota Watanabe, Ken Ishii, Yoshiaki Toyama, Keiyo Takubo, Keisuke Horiuchi, Takeshi Miyamoto, Masaya Nakamura, Morio Matsumoto

**Affiliations:** Department of Orthopaedic Surgery, Keio University School of Medicine, 35 Shinanomachi, Shinjyuku-ku, Tokyo 160-8582 Japan; Department of Orthopaedic Surgery, National Defense Medical College, 3-2 Namiki, Tokorozawa, Saitama, 359-8513 Japan; Department of Stem Cell Biology, Research Institute, National Center for Global Health and Medicine, 1-21-1 Toyama, Shinjuku-ku, Tokyo, 162-8655 Japan

**Keywords:** Intervertebral disc degeneration, Oxidative stress, Reactive oxygen species, Antioxidant

## Abstract

**Introduction:**

Oxidative stress has been reported to be involved in numerous human diseases, including musculoskeletal disorders such as osteoarthritis. However, the interaction between intervertebral disc (IVD) degeneration and oxidative stress is not well understood. The purpose of the present study was to elucidate the contribution of oxidative stress to IVD degeneration and the efficacy of antioxidant treatment for degenerative discs.

**Methods:**

The expression level of an oxidative stress marker, nitrotyrosine, was assessed by immunohistochemistry and Western blotting. For evaluating intracellular reactive oxygen species (ROS) levels and oxidative stress in rat annulus fibrosus (AF) cells, flow cytometry and luciferase assay with an OKD48 construct were performed. The grade of IVD degeneration was assessed by magnetic resonance imaging and histological analysis.

**Results:**

A high frequency of nitrotyrosine-positive cells was observed in rat and human degenerative discs. mRNA expression of catabolic factors such as tumor necrosis factor-alpha (TNF-alpha), matrix metalloprotease-3 (MMP-3), and cyclooxygenase-2 (COX-2) was significantly induced by treatment with H_2_O_2_ or buthionine sulfoximine, whereas that of aggrecan, an important chondrogenic proteoglycan, was reduced in a dose-dependent manner. Treatment with mitogen-activated protein kinase (MAPK) inhibitors blocked the inductive effect of excessive ROS on COX-2 mRNA expression. Western blotting confirmed the phosphorylation of MAPKs in H_2_O_2_ and BSO-treated AF cells. Conversely, we showed that TNF-α induced oxidative stress with increased intracellular ROS levels in AF cells. Treatment with the antioxidant N-acetyl cysteine (NAC) abrogated the catabolic effect of excessive ROS and TNF-alpha *in vitro*. Finally, we showed that oral administration of NAC prevented IVD degeneration in rat degenerative model.

**Conclusions:**

A positive feedback loop was formed between excessive ROS and TNF-alpha in AF cells. Thus, oxidative stress contributes to the progression of IVD degeneration and NAC can be a therapeutic option for IVD degeneration.

**Electronic supplementary material:**

The online version of this article (doi:10.1186/s13075-015-0834-8) contains supplementary material, which is available to authorized users.

## Introduction

Intervertebral disc (IVD) degeneration is clinically related to chronic low back pain, disc herniation, spinal canal stenosis, and spinal deformities [1]. These spinal disorders are the leading causes of disability in the workforce and result in large economic and social costs [2]. The etiology of IVD degeneration is complex and multifactorial, with contributions from aging, mechanical stress, smoking, infection, trauma, and heredity [3–5]. At present, the molecular mechanisms underlying IVD degeneration are largely unclear, and there are no effective therapies.

IVDs, which lie between the adjacent vertebral bodies and provide flexibility and load support in the spine, are composed of two discrete components: the nucleus pulposus (NP) and the annulus fibrosus (AF). The interior structure, the NP, is an avascular cartilage-like tissue that contains extracellular matrix (ECM) proteins rich in proteoglycans. The AF, a fibrous cartilage composed of an inner and outer coaxial lamella, enwraps the NP. The NP has two different cell populations in humans: small chondrocyte-like cells and large vacuolated notochordal cells [6–8]. Because notochordal cells are rarely present after adolescence, the pathogenesis of IVD degeneration is possibly linked to loss of the cells [6, 9].

IVD degeneration is characterized by the reduction of water content and ECM breakdown. Biologically, this degeneration represents a loss of steady-state metabolism that probably results from an imbalance between anabolic and catabolic processes [10, 11]. Increased expression of proinflammatory cytokines such as interleukin (IL)-1 and tumor necrosis factor alpha (TNFα) and loss of aggrecan, a major component of proteoglycan in IVD, have been observed in degenerative discs [12, 13]. These changes are associated with the increased expression of matrix-degrading enzymes such as matrix metalloproteases (MMPs) and a disintegrin and metalloprotease with thrombospondin motifs (ADAMTS) [14–17]. Many studies have found elevated levels of MMP-1, MMP-2, MMP-3, and MMP-13 in degenerated IVD [14, 15]. In addition, several lines of evidence support the presence of prostaglandins in IVD under stress and implicate the possible involvement of prostaglandins in the progression of IVD degeneration [18, 19]. The expression of cyclooxygenase-2 (COX-2), a key enzyme in prostaglandin biosynthesis in disc cells, has also been shown to be induced by mechanical stress, another predisposing factor that can disrupt the disc structure and initiate the degenerative cascade [20, 21]. These inflammatory reactions are clinically known to be the cause of lower back pain or radiculopathy in spinal disorders [22].

According to the free-radical theory of aging, oxidative stress initiated by reactive oxygen species (ROS) contributes to the functional decline that is characteristic of aging [23]. ROS, including the superoxide anion (O^2−^), the hydroxyl radical (OH), hydrogen peroxide (H_2_O_2_), and nitric oxide (NO), all of which can diffuse through membranes, are byproducts of cellular oxidative metabolism. Excessive ROS can overwhelm the antioxidant scavenging capacity within a cell and cause oxidative damage to DNA, lipids, and proteins as well as concomitant cellular damage. Numerous diseases are known to involve oxidative stress, including musculoskeletal diseases such as osteoarthritis and osteoporosis [24–26]. In IVD, the expression level of similar oxidative stress markers such as carboxymethyl-lysine, advanced glycation end products (AGEs), and peroxynitrite were reported to be elevated in degenerative human discs [27–29]. In cultured disc cells, the activation of p38 mitogen-activated protein kinase (MAPK), extracellular signal-regulated kinase (ERK), c-Jun N-terminal kinase (JNK), and Akt signaling pathways and nuclear translocation of nuclear factor (NF)-κB and Nrf2 were reported to be induced by treatment with H_2_O_2_ [30]. In vivo, the mitochondria-targeted ROS scavenger rescued age-related disc degeneration in the murine model [31]. However, the interaction between IVD degeneration and oxidative stress induced by excessive ROS is not yet completely understood. The primary objective of this study was to clarify the role of excessive ROS in the catabolic cascade of degenerative IVD. The secondary goal was to determine whether excessive ROS represents a therapeutic target for IVD degeneration. We clearly demonstrated that oxidative stress contributes to IVD degeneration and that antioxidant treatment rescues the phenotype of the IVD degeneration model. These results indicate that excessive ROS plays an important role in the pathogenesis of disc disease and can offer a therapeutic target to treat this debilitating and painful degenerative condition.

## Methods

### Human samples

For the experimental use of surgical samples, written informed consent was obtained from each patient according to the Keio University (Tokyo, Japan) Hospital Ethics Guideline (Keio Hospital #15-52). A total of 10 IVD tissues were dissected from patients with degenerative disease, including eight with degenerative lumbar scoliosis, one with degenerative lumbar kyphosis, and one with adult idiopathic scoliosis. They were evaluated according to Pfirrmann’s magnetic resonance classification (Table 1). As a nondegenerative disc, 17-year-old male IVD dissected at autopsy was used. They were fixed in 4 % paraformaldehyde in phosphate-buffered saline and embedded in paraffin to obtain sections 4 μm thick.

**Table 1:** Patient details used for the experiments

Sample number	Sex	Age (years)	Level	Grading^a^	Diagnosis
1	Female	32	L1/2	3	Adult idiopathic scoliosis
2	Male	55	L2/3	3	Degenerative lumber scoliosis
3	Male	55	L3/4	3	Degenerative lumber scoliosis
4	Female	53	L3/4	3	Degenerative lumber scoliosis
5	Female	53	L2/3	4	Degenerative lumber scoliosis
6	Female	75	L1/2	4	Degenerative lumber scoliosis
7	Female	75	L2/3	4	Degenerative lumber scoliosis
8	Female	75	L3/4	5	Degenerative lumber scoliosis
9	Female	75	L4/5	5	Degenerative lumber scoliosis
10	Male	77	L2/3	5	Degenerative lumber kyphosis

^a^Pfirrmann disc degeneration grading

### Animal model of IVD degeneration

All animals were purchased from Japan Clea (Tokyo, Japan) or born and kept under pathogen-free conditions and were cared for in accordance with the guidelines of the Keio University School of Medicine. Posterior incision above the coccyx IVD of 8-week-old female Wistar rats was made, and the soft tissues such as posterior tendons and ligaments were separated under anesthesia. Disc puncture was performed using a 23-gauge needle on the 3rd–10th coccygeal vertebrae, as in a previous study [32, 33]. For analysis, a total of 27 rats were sacrificed. A total of nine IVDs were used in each group. For real-time RT-PCR analysis, AF tissues were dissected through a microscope and homogenized only once 1 week after the puncture. The total RNA of the homogenized AF was extracted using TRIzol Reagent (Cosmo Bio Company, Tokyo, Japan). For western blotting, the protein of AF tissues was extracted using Tissue Protein Extraction Reagent (TPER) 1 month after puncture. Magnetic resonance imaging (MRI) was performed 2 months after the puncture. After that, AF tissues were dissected for histological analysis. The harvested discs were decalcified and embedded in paraffin, and sections 4 μm thick were cut. To assess the effect of antioxidant treatment, *N*-acetyl cysteine (NAC, 1 g/l; Sigma-Aldrich, St. Louis, MO, USA) was given orally to degenerative model rats 1 week before puncture and continued for another 2 months until MRI and histological analysis.

### Imaging

Mid-sagittal T2-weighted MRI (TR/TE, 3000/86; echo train length, 12; slice thickness, 2 mm; field of view, 10 cm; matrix size, 352 × 224; number of excitations, 10 times) (GE Sigma Excite HD 1.5 T; GE Healthcare, Tokyo, Japan) of rat IVD was performed. The ratio of the high-intensity area to IVD was measured using Image Processing and Analysis in Java (Oracle Corporation, Redwood Shores, CA, USA).

### Histology

Deparaffinized sections of rat IVD were stained with hematoxylin and eosin (H&E). The sections were stained with anti-nitrotyrosine antibody (diluted 100-fold; Abcam, Cambridge, UK), followed by staining with horseradish peroxidase (HRP)-conjugated goat antimouse IgG (diluted 200-fold; Sigma-Aldrich), anti-TNFα antibody (diluted 100-fold; Novus Biologicals, Littleton, CO, USA), and anti-IL-1β (diluted 100-fold; Bioworld Technology, St. Louis Park, MN, USA), followed by HRP-conjugated goat antirabbit IgG (diluted 200-fold; Sigma-Aldrich). Staining was visualized using diamino benzidine (nacalai tesque, Kyoto, Japan). Nuclei were stained with hematoxylin. Antigen retrieval was achieved by pressure-cooking in citrate buffer (pH 6.0) for 20 minutes. All specimens were viewed under a microscope (BZ-9000; Keyence Co., Osaka, Japan). The frequency of nitrotyrosine-positive cells was measured using Image Processing and Analysis in Java at a magnification of × 200. We chose three individual areas at random and calculated the average of the frequency.

### Isolation and culture of AF cells

Rat AF tissues were macroscopically dissected from the lumbar and coccyx IVD of 8-week-old female Wistar rats and digested using pronase E (0.04 %) (SERVA, Heidelberg, Germany) for 1 hour at 37 °C and collagenase P (0.025 %) (Roche Diagnosis, Tokyo, Japan) for 4 hours at 37 °C. The cells were then washed with Dulbecco’s modified Eagle’s medium (DMEM; Invitrogen, Carlsbad, CA, USA) containing 5 % heat-inactivated fetal bovine serum (FBS; JRH Biosciences, Lenexa, KS, USA) according to a previous method [34]. The isolated AF cells were cultured in DMEM supplemented with 10 % FBS and 1 % penicillin–streptomycin, maintained in a humidified incubator containing 5 % CO_2_ at 37 °C, and used within the first five passages for in vitro analysis. We have confirmed there was no obvious difference in the mRNA expression level of important ECM of AF cells, type I collagen, type II collagen, and aggrecan between the second and fifth passaged cells (Additional file 1: Figure S1).

### Treatment of oxidative stress, TNFα, MAPK inhibitors, and antioxidants

AF cells were treated with H_2_O_2_ (0, 10, 100 μM) (Wako, Tokyo, Japan), buthionine sulfoximine (BSO; 0, 0.2, 1 mM) (Sigma-Aldrich), and TNFα (50 ng/ml) (R&D Systems, Minneapolis, MN, USA) for 24 hours. For analysis of phosphorylation, AF cells were treated with 100 μM H_2_O_2_ for 0, 5, 10, 15, 30, or 60 minutes and 50 ng/ml TNFα for 10 minutes. Cells were pretreated with MAPK signaling inhibitors for 30 minutes followed by incubation with H_2_O_2_ or BSO for 24 hours. In this assay, we utilized p38 inhibitor (SB203580, 10 μmol/l), JNK inhibitor (SP600125, 10 μmol/l), or ERK inhibitor (PD98059, 10 μmol/l). All inhibitors were purchased from Wako. To study the effect of antioxidative agents, H_2_O_2_-treated, BSO-treated, or TNFα-treated AF cells were cultured with 100 μM NAC and 20 mM α-tocopherol (Wako) solved in ethanol for 15 minutes, 30 minutes, or 24 hours. In the case of α-tocopherol experiment, ethanol was added at the same concentration to ROS-mediated or TNFα-treated AF cells. The experiment of α-tocopherol treatment was independently carried out five times, and all other experiments were independently carried out three times.

### Real-time RT-PCR

Total RNA was isolated from AF cells using the RNeasy Mini Kit (Qiagen GmbH, Hilden, Germany) or TRIzol Reagent. First-strand cDNA was prepared using the Prime Script RT Reagent Kit (Takara Bio, Shiga, Japan) according to the manufacturer’s instructions. Real-time RT-PCR was performed using the Thermal Cycler Dice Real-Time System and SYBR Premix Ex Taq (Takara Bio), and the results were quantified using the ddCt method. We measured the relative mRNA expression of TNFα, MMP-3, COX-2, and aggrecan normalized by the expression of β-actin or hypoxanthine phosphoribosyl transferase (HPRT). Gene-specific forward and reverse primers were as follows: *β-actin*-forward, 5′-TGAGAGGGAAATCGTGCGTGAC-3′; *β-actin*-reverse, 5′-AAGAAGGAAGGCTGGAAAAGAG-3′; *HPRT*-forward, 5′-TCCTCATGGACTGATTATGGACA-3′; *HPRT*-reverse, 5′-TAATCCAGCAGGTCAGCAAAGA-3′; *TNFA*-forward, 5′-GCAGATGGGCTGTACCTTATC-3′; *TNFA*-reverse, 5′-GGCTGACTTTCTCCTGGTATG-3′; *MMP3*-forward, 5′-GGACCAGGGATTAATGGAGATG-3′; *MMP3*-reverse, 5′-TGAGCAGCAACCAGGAATAG-3′; *COX2*-forward, 5′-TGAACACGGACTTGCTCACTTTG-3′; *COX2*-reverse, 5′-AGGCCTTTGCCACTGCTTGTA-3′; *AGC1*-forward, 5′-GGATCTATCGGTGTGAAGTGATG-3′; *AGC1*-reverse, 5′-AGTGTGTAGCGTGTGGAAATAG-3′; *Col1a1*-forward, 5′-AGCTCCTGGGCCTATCTGATGA-3′; *Col1a1*-reverse, 5′-AATGGTGCTCTGAAACCCTGATG-3′; *Col2a1*-forward, 5′-GAGGGCAACAGCAGGTTCAC-3′; and *Col2a1*-reverse, 5′-GCCCTATGTCCACACCAAATTC-3′.

### Intracellular ROS

The dissociated cells were loaded with Mitotracker Orange CMH2TM ROS (Life Technologies, Carlsbad, CA, USA) and incubated on a shaker at 37 °C for 30 minutes. For the estimation of intracellular ROS, a FACS Calibur was used (Becton-Dickinson Immunocytometry Systems, San Jose, CA, USA).

### Western blotting

Total cell protein was extracted using a mammalian protein extraction reagent (Thermo Fisher Scieintific, Waltham, MA, USA). All of the wash buffers and extraction buffers included 1× protease inhibitor cocktail (Roche), NaF (1 M), and Na_3_VO_4_ (50 μM). Cell lysates mixed with loading buffer (Tris–Glycine SDS Sample Buffer (Invitrogen) with 5 % 2-mercaptoethanol) were loaded onto 15 % polyacrylamide gels and electrophoresed by sodium dodecyl sulfate-polyacrylamide gel electrophoresis. Proteins were transferred to polyvinylidenefluoride membranes (ATTO Corporation, Tokyo, Japan). The membranes were blocked using 5 % nonfat dry milk in Tris-buffered saline containing 0.1 % Triton X-100 (TBST) (50 mM Tris, pH 7.6, 150 mM NaCl, 0.1 % Tween 20) and incubated overnight at 4 °C in 3 % nonfat dry milk in TBST with antibody against p38 (1:1000; Cell Signaling Technology, Boston, MA, USA), phosphorylated p38 (1:1000; Cell Signaling Technology), ERK (1:1000; Cell Signaling Technology), phosphorylated ERK (1:2000; Cell Signaling Technology), JNK (1:1000; Cell Signaling Technology), phosphorylated JNK (1:1000; Cell Signaling Technology), NF-κB p65 (1:1000; Cell Signaling Technology), phosphorylated NF-κB p65, and β-actin (1:1000; Cell Signaling Technology), followed by HRP-conjugated goat antirabbit IgG (1:2000; Sigma-Aldrich). Proteins were visualized using ECL Western Blotting Detection Reagent (GE Healthcare, Uppsala, Sweden).

### Transfections and dual luciferase assay

Cells were transferred to 24-well plates at a density of 5 × 10^4^ cells/well 1 day before transfection. LipofectAMINE 3000 (Invitrogen) was used as the transfection reagent. The experiment was performed following the manufacturer’s recommendation. The optimized ratio of plasmids and posttransfection period were determined in previous reports [35]. Cells were cotransfected with 300 ng GL4-F (OKD48) with 200 ng pRL-TK plasmid. Plasmids were premixed with the transfection reagent for each transfection. Transfected cells were harvested the next day, and the Dual-Luciferase Reporter Assay System (Promega, Madison, WI, USA) was used for sequential measurements of firefly and *Renilla* luciferase activities. Luciferase activities were quantified, and relative ratios were calculated using a luminometer (TD-20/20; Turner Designs, Sunnyvale, CA, USA). Three independent transfections were performed, and all analyses were carried out in triplicate.

### Plasmids

P(3 × ARE)TKbasal-hNrf2(1–433)-GL4-F (OKD48) was provided by Dr Takao Iwawaki, Gunma University [36]. The vector pRL-TK (Promega) harboring the *Renilla reniformis* luciferase gene was used as an internal transfection control.

### Statistical analysis

All measurements were performed in triplicate. Data are presented as mean ± standard deviation (SD). Differences between the groups were analyzed by Student’s *t* test and analysis of variance (ANOVA). *p* <0.05 was considered to indicate statistical significance.

## Results

### Induction of ROS level in IVD degeneration

**Fig. 1:**
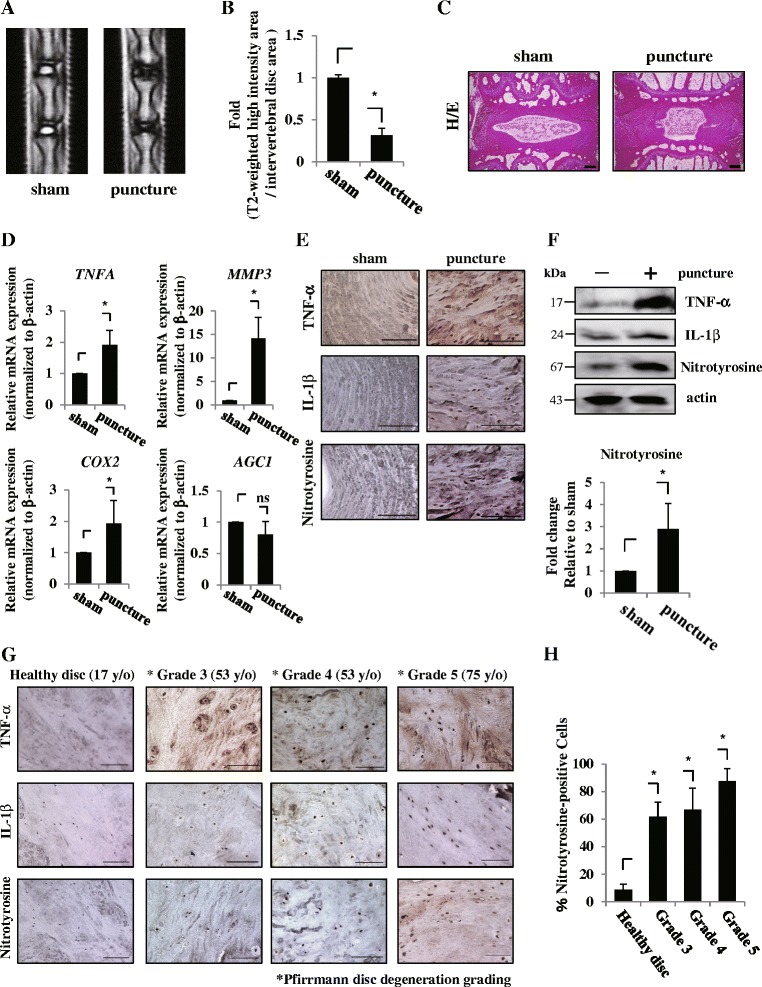
Induction of ROS level in IVD degeneration. **a** Mid-sagittal T2-weighted MRI findings of IVDs 2 months after the puncture confirm lower signal intensity than that of the sham group. **b** Compared with the sham group, the ratio of the high-intensity area to IVD areas was significantly decreased by needle puncture. **c** Hematoxylin and eosin (*H/E*) staining shows smaller NP and less-organized lamellae of AF in the punctured group. Scale bars, 300 μm. **d** Real-time RT-PCR analysis. mRNA expression of TNFα, MMP-3, and COX-2 was significantly induced in the AF of the punctured model, whereas that of aggrecan tended to be reduced. Data presented as mean ± SD of three independent experiments performed in triplicate (*n* = 3). **e** Numbers of nitrotyrosine-positive cells were increased in AF of the punctured model, correlated with elevated expression of TNFα and IL-1β. Scale bars, 100 μm. **f** Western blotting and densitometry analysis. The protein expression level of nitrotyrosine was significantly higher as well as TNFα and IL-1β in the rat degenerative model. **g** Immunohistochemistry of human degenerative disc samples with each grade including healthy disc. Scale bars, 100 μm. **h** More than grade 3 degenerative discs had significantly higher frequency of nitrotyrosine-positive cells compared with healthy disc. *p <0.05. *COX* cyclooxygenase, *IL* interleukin, *MMP* matrix metalloprotease, *ns* not significant, *TNF* tumor necrosis factor

To investigate the involvement of ROS in IVD degeneration, we first assessed one of the oxidative stress markers, nitrotyrosine, in a rat punctured model and in human degenerative IVD samples. A total of nine discs per group were used for the analysis. Mid-sagittal T2-weighted MRI findings of IVD in the rat punctured model confirmed a lower signal intensity than that in the sham group (Fig. 1a). The ratio of the high-intensity area to IVD was significantly reduced in the model (Fig. 1b). H&E staining showed a smaller NP and less-organized lamellae of AF in the punctured model (Fig. 1c). In addition, we confirmed that the mRNA expression of TNFα, MMP-3, and COX-2—catabolic molecules involved in degeneration—was significantly induced in AF of the punctured model, whereas that of aggrecan tended to be reduced, but not significantly (Fig. 1d). These results suggest that the needle punctured model is adequate for analysis of IVD degeneration. The expression of nitrotyrosine as well as TNFα and IL-1β was higher in AF of this model group compared with that of the sham group (Fig. 1e). Western blotting also showed a higher protein expression level of nitrotyrosine as well as TNFα and IL-1β in the rat degenerative model (Fig. 1f). Densitometry analysis confirmed these observations (Fig. 1f). Moreover, immunohistochemistry showed that human degenerative disc samples with each grade had a high proportion of nitrotyrosine-positive cells, accompanied by robust expression of TNFα and IL-1β, whereas human healthy disc had low expression of these markers (Fig. 1g). We assessed the frequency of nitrotyrosine-positive cells in each grade sample. Figure 1h showed that more than grade 3 degenerative discs had a significantly higher frequency of nitrotyrosine-positive cells compared with healthy disc (Fig. 1h).

### Molecular phenotype of the rat AF cells treated with ROS

**Fig. 2:**
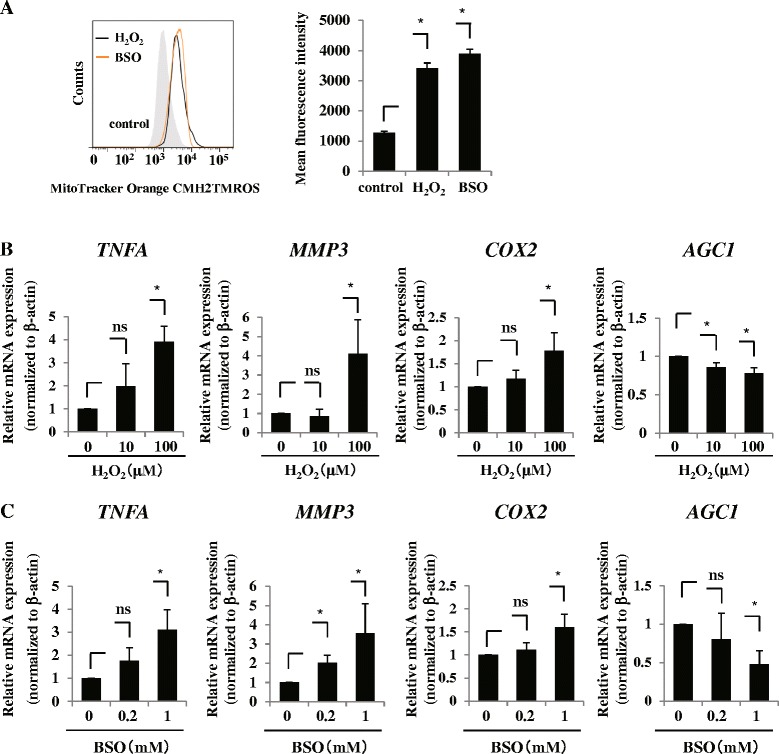
Molecular phenotype of the rat AF cells treated with ROS. **a** Flow cytometry confirmed that the intracellular level of ROS was significantly increased by treatment with H_2_O_2_ and BSO in AF cells. **b** Real-time RT-PCR analysis showed that the mRNA expression of TNFα, MMP-3, and COX-2 was significantly induced by treatment with H_2_O_2_, whereas aggrecan mRNA expression was reduced in a dose-dependent manner. **c** mRNA expression of TNFα, MMP-3, and COX-2 was significantly induced by treatment with BSO, whereas aggrecan mRNA expression was reduced in a dose-dependent manner, as determined by real-time RT-PCR. Data presented as mean ± SD of three independent experiments performed in triplicate (*n* = 3); *p <0.05. *BSO* buthionine sulfoximine, *COX* cyclooxygenase, *H*_*2*_ *O*_*2*_ hydrogen peroxide, *MMP* matrix metalloprotease, *ns* not significant, *TNF* tumor necrosis factor

**Fig. 3:**
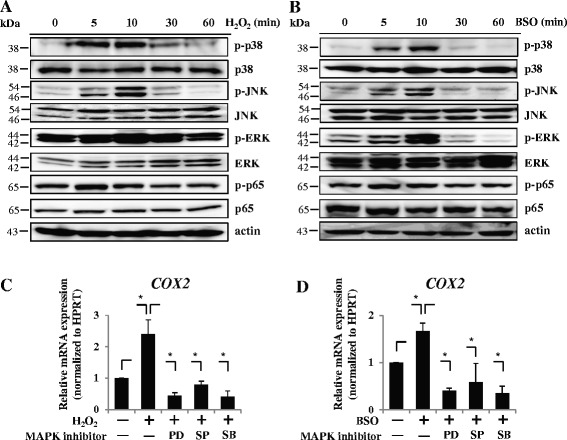
Downstream signaling of ROS in AF cells. **a**, **b** Western blot analysis showed that mitogen-activated protein kinases (*MAPKs*), including p38, ERK, and JNK, were maximally phosphorylated 10 minutes after treatment with H_2_O_2_ **a** and BSO **b**. **c**, **d** Real-time RT-PCR analysis. Treatment of AF cells with MAPK signaling inhibitors, including p38 inhibitor (*SB*), JNK inhibitor (*SP*), and ERK inhibitor (*PD*), significantly abolished H_2_O_2_-mediated **c** or BSO-mediated **d** induction of COX-2 mRNA expression. Data presented as mean ± SD of three independent experiments performed in triplicate (*n* = 3); *p <0.05. *BSO* buthionine sulfoximine, *COX* cyclooxygenase, *ERK* extracellular signal-regulated kinase, *H*_*2*_ *O*_*2*_ hydrogen peroxide, *HPRT* hypoxanthine phosphoribosyl transferase, *JNK* c-Jun N-terminal kinase

To clarify the pathophysiological role of intracellular ROS, we examined the phenotype of the AF cells treated with H_2_O_2_ and BSO, which is a glutathione synthesis inhibitor that activates oxidative stress. Flow cytometry confirmed that the intracellular level of ROS was significantly increased by treatment with both H_2_O_2_ and BSO in AF cells (Fig. 2a). Next, we treated rat cultured AF cells with H_2_O_2_ and evaluated the expression of catabolic and anabolic factors of IVD degeneration by real-time RT-PCR analysis. We found that the mRNA expression of TNFα, MMP-3, and COX-2 was significantly induced, whereas that of aggrecan was reduced in a dose-dependent manner (Fig. 2b). Expectedly, real-time RT-PCR showed similar results with BSO treatment (Fig. 2c). To investigate the downstream signaling of ROS in AF cells, we evaluated the phosphorylation of MAPKs, including p38, ERK, and JNK, as well as p65 by western blotting. This analysis showed that the three signaling pathways of MAPK were maximally phosphorylated 10 minutes after treatment with H_2_O_2_ and BSO (Fig. 3a, b). On the other hand, the phosphorylation of p65 was not activated by their treatments (Fig. 3a, b). Next, to investigate further the involvement of MAPKs, we cultured H_2_O_2_-treated or BSO-treated AF cells with MAPK signaling inhibitors, including p38 inhibitor (SB203580), JNK inhibitor (SP600125), and ERK inhibitor (PD98059), and assessed the mRNA expression of COX-2, TNFα, and MMP-3 by real time RT-PCR analysis. Figure 3c, d shows that all inhibitors significantly abolished H_2_O_2_-mediated or BSO-mediated induction of COX-2 mRNA expression. These results suggest that the catabolic effect of excessive ROS is mediated through the signaling pathways of p38, ERK, and JNK in AF cells. However, the results of TNFα and MMP-3 did not clearly show the effect of these inhibitors compared with COX-2 (Additional file 2: Figure S2). The gene regulatory network of TNFα and MMP3 can be more complicated than that of COX-2. The experiment concerned with the mechanisms of the difference is in progress.

### Antioxidant significantly neutralized the catabolic effect of ROS

**Fig. 4:**
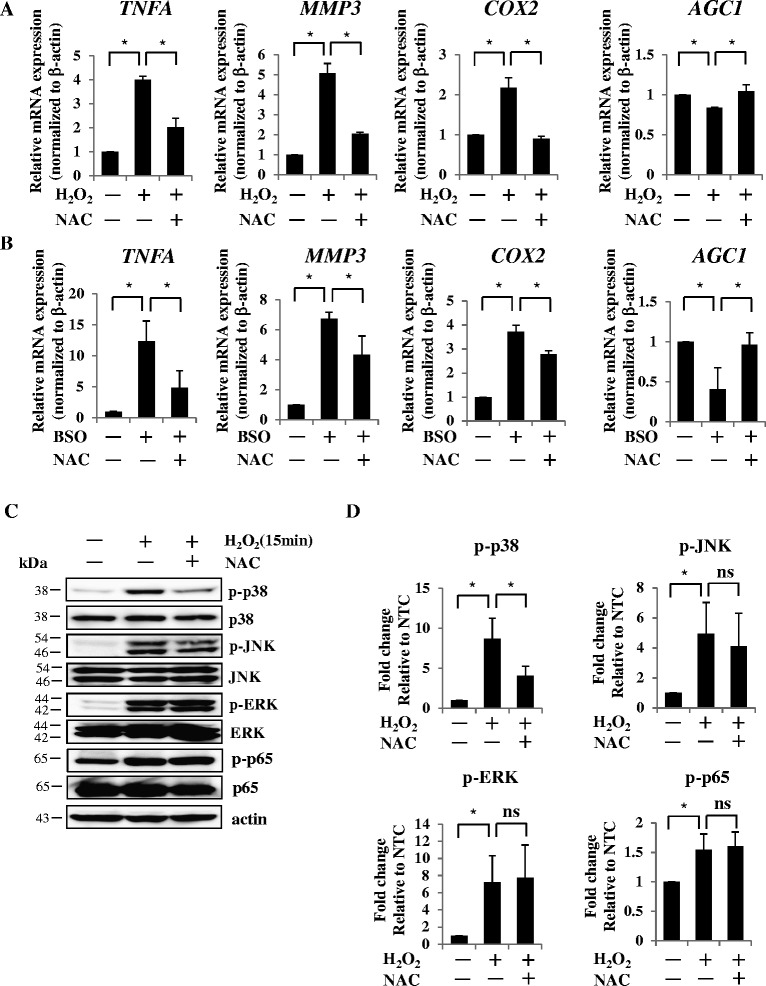
NAC significantly neutralizes the catabolic effect of ROS. **a**, **b** NAC significantly abolished the induction of TNFα, MMP-3, and COX-2 expression and reduction of aggrecan expression in H_2_O_2_-treated **a** or BSO-treated **b** AF cells by real-time RT-PCR analysis. **c**, **d** Western blotting and densitometry clearly showed that NAC treatment inhibited the phosphorylation of p38 but not of JNK, ERK, and p65. NTC, nontreated control. Data presented as mean ± SD of three independent experiments performed in triplicate (*n* = 3); *p <0.05. *BSO* buthionine sulfoximine, *COX* cyclooxygenase, *ERK* extracellular signal-regulated kinase, *H*_*2*_ *O*_*2*_ hydrogen peroxide, *JNK* c-Jun N-terminal kinase, *MMP* matrix metalloprotease, *NAC* N-acetyl cysteine, *ns* not significant, *TNF* tumor necrosis factor

**Fig. 5:**
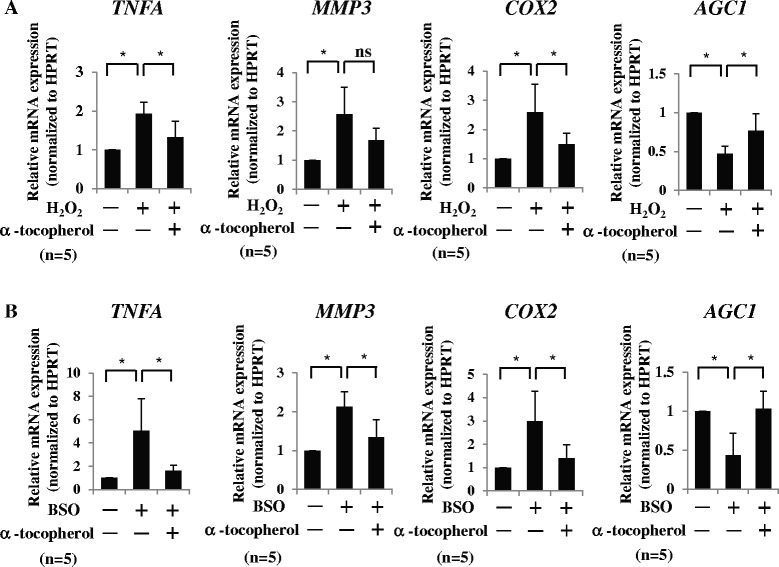
Treatment of α-tocopherol significantly abolishes the catabolic effect of ROS. **a**, **b** Real-time RT-PCR analysis. Treatment with α-tocopherol significantly abolished the H_2_O_2_-mediated **a** or BSO-mediated **b** induction of TNFα and COX-2 mRNA expression, and reduction of aggrecan. H_2_O_2_-mediated induction of MMP-3 expression was not significant, but tended to be decreased. Data presented as mean ± SD of five independent experiments performed in triplicate (*n* = 5); *p <0.05. *BSO* buthionine sulfoximine, *COX* cyclooxygenase, *H*_*2*_ *O*_*2*_ hydrogen peroxide, *HPRT* hypoxanthine phosphoribosyl transferase, *MMP* matrix metalloprotease, *ns* not significant, *TNF* tumor necrosis factor

Next, to ascertain whether the treatment of antioxidant attenuates the ROS-mediated catabolic effect in AF cells, we treated AF cells with NAC, together with H_2_O_2_ or BSO. Using real-time RT-PCR analysis, we found that NAC significantly abolished the induction of TNFα, MMP-3, and COX-2 expression and reduction of aggrecan expression in H_2_O_2_-treated or BSO-treated AF cells (Fig. 4a, b). To investigate whether NAC regulates the downstream signaling of ROS in AF cells, we assessed the phosphorylation of p38, ERK, and JNK in AF cells treated with and without NAC. Western blotting and densitometry analysis clearly showed that treatment with NAC inhibited the phosphorylation of p38 but not of JNK, ERK, and p65 (Fig. 4c, d). We also elucidated whether the other antioxidant material α-tocopherol (vitamin E) could replicate these effects. As expected, real-time RT-PCR analysis showed that treatment with α-tocopherol also abolished the H_2_O_2_-mediated or BSO-mediated change of TNFα, COX-2, MMP-3, and aggrecan mRNA expression (Fig. 5a, b).

### Treatment with antioxidant also significantly inhibited the effect of TNFα on AF cells

**Fig. 6:**
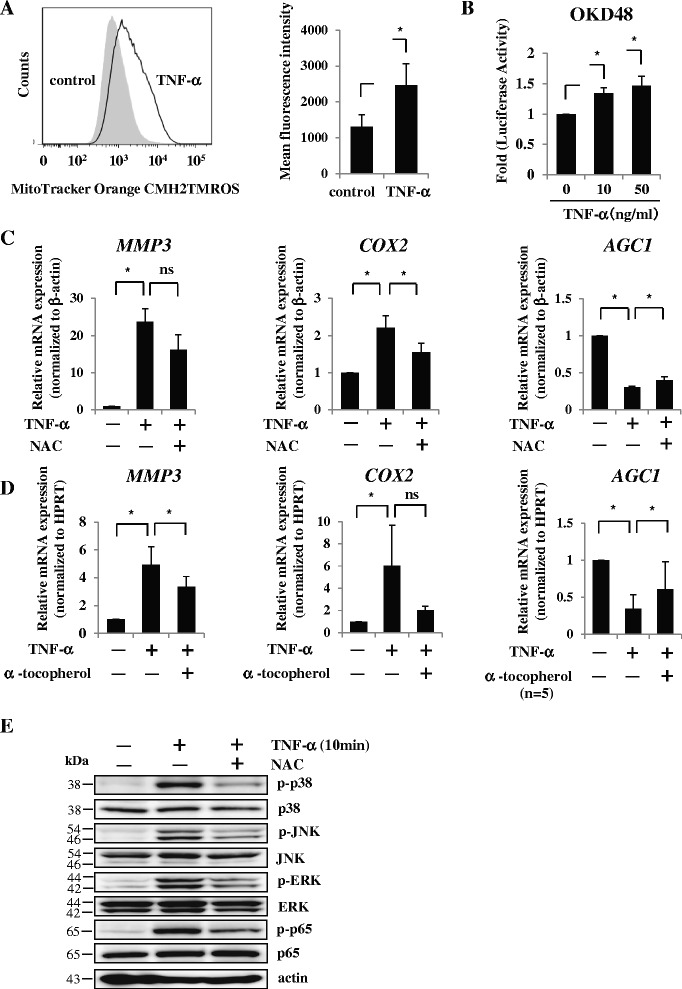
Catabolic effect of TNFα is partially mediated by ROS in AF cells. **a** Flow cytometry showed that ROS levels were significantly increased in AF cells after treatment with TNFα. **b** Reporter activity of the OKD48 construct was significantly induced by treatment with TNFα (10 and 50 ng/ml) in AF cells. **c** Real-time RT-PCR analysis. Treatment of NAC significantly attenuated TNFα-mediated induction of COX-2 mRNA expression and reduction of aggrecan in AF cells. Data presented as mean ± SD of three independent experiments performed in triplicate (*n* = 3). **d** α-Tocopherol significantly reduced MMP-3 mRNA expression and restored aggrecan mRNA expression in the TNFα-treated cells. Data presented as mean ± SD of five independent experiments performed in triplicate (*n* = 5). **e** Western blotting. NAC treatment inhibited the phosphorylation of p38, ERK, JNK, and p65 in TNFα-treated AF cells. *p <0.05. *COX* cyclooxygenase, *ERK* extracellular signal-regulated kinase, *HPRT* hypoxanthine phosphoribosyl transferase, *JNK* c-Jun N-terminal kinase, *MMP* matrix metalloprotease, *NAC* N-acetyl cysteine, *ns* not significant, *TNF* tumor necrosis factor

To assess whether intracellular ROS levels are regulated by inflammatory cytokines in AF cells, we treated cultured AF cells with TNFα and evaluated the level of intracellular ROS using MitoTracker Orange CMH2TMR. Flow cytometry showed that ROS levels were significantly increased in AF cells after treatment with TNFα (Fig. 6a). Recently, Oikawa et al. [36] reported that an OKD48 construct (P(3 × ARE)TKbasal-hNrf2(1–433)-GL4-F) specifically responded to oxidative stress and was useful for monitoring stress in vitro and in vivo. We transfected the plasmids to AF cells and assessed the reporter activity with and without TNFα. As expected, the activity was significantly induced by treatment with TNFα in AF cells (Fig. 6b). These results suggest that TNFα upregulates the intracellular ROS level and induces oxidative stress in AF cells. In addition, real-time RT-PCR showed that treatment with NAC attenuated TNFα-mediated induction of MMP-3 and COX-2 expression and reduction of aggrecan in AF cells (Fig. 6c). In the case of α-tocopherol, significant abolishment of TNFα-mediated induction of MMP-3 and reduction of aggrecan was also observed. Concerning COX-2, α-tocopherol had the tendency to attenuate, but not significantly, the influence of TNFα (Fig. 6d). Moreover, western blotting clearly showed that NAC treatment inhibited the phosphorylation of p38, ERK, JNK, and p65 in TNFα-treated AF cells (Fig. 6e). These results indicated that in AF cells, the catabolic influence of TNFα was partly mediated by ROS signaling.

### Oral administration of NAC improved IVD degeneration in a rodent model

**Fig. 7:**
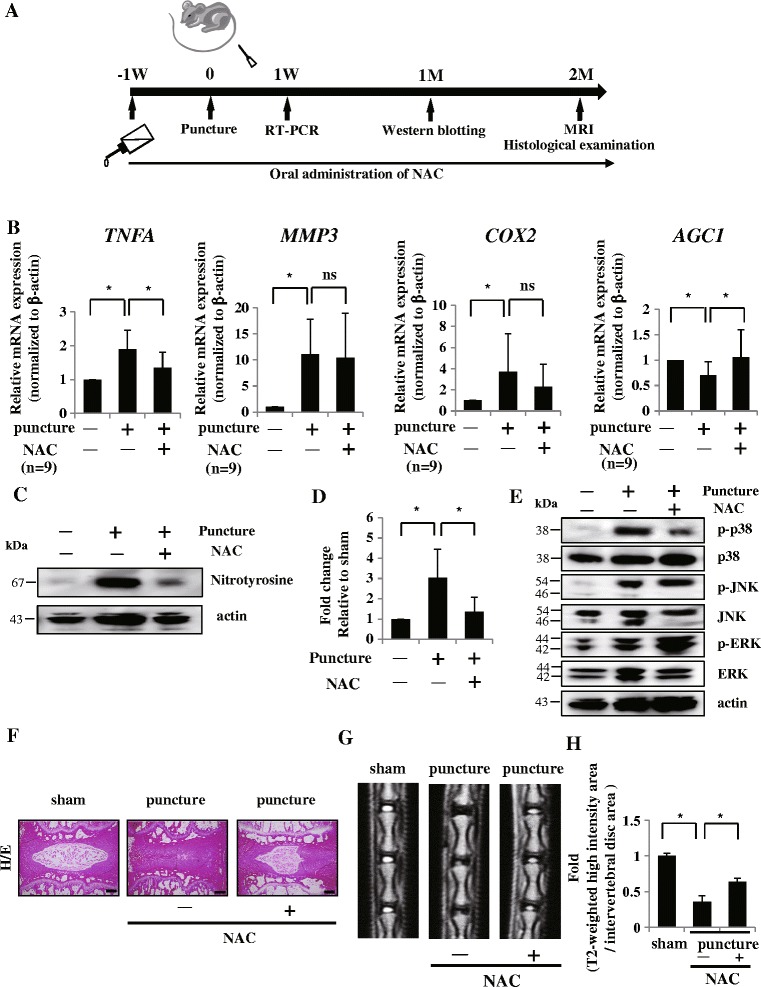
Oral NAC administration improves IVD degeneration in the rodent model. **a** Schematic representation of oral NAC administration experiment for IVD degenerative model. **b** Real-time RT-PCR analysis. NAC significantly abolished the induction of TNFα mRNA expression and reduction of aggrecan in punctured AF tissues. Data presented as mean ± SD of nine IVD discs derived from three independent rats performed in triplicate (*n* = 9); *p <0.05. **c** Western blotting. Protein expression of nitrotyrosine was clearly reduced by oral administration of NAC. **d** Densitometry confirmed that the protein level of nitrotyrosine was significantly abrogated by oral administration of NAC. **e** Western blotting. Phosphorylation of p38 was significantly attenuated by treatment of NAC. **f** Histological examination of H&E staining showed that oral administration of NAC improved the reduction of NP size and the disorganization of the AF in this degenerative model. Scale bars, 300 μm. **g** Mid-sagittal T2-weighted MRI. Area of T2 high intensity was increased by oral administration of NAC in the degenerative group. Representative data are shown. **h** The T2-weighted high-intensity area to IVD ratio was significantly improved by NAC administration. Data presented as mean ± SD; *p <0.05. *COX* cyclooxygenase, *ERK* extracellular signal-regulated kinase, *H/E* hematoxylin and eosin, *JNK* c-Jun N-terminal kinase, *MMP* matrix metalloprotease, *MRI* magnetic resonance imaging, *NAC* N-acetyl cysteine, *ns* not significant, *TNF* tumor necrosis factor

Finally, to investigate the efficacy of NAC on IVD degeneration in vivo, we administered NAC (1 g/l) orally to degenerative model rats 1 week before puncture and continued for another 1 week until RNA isolation (*n* = 9), for 1 month until protein isolation (*n* = 9), and for 2 months until MRI analysis and histological examination (*n* = 9) (Fig. 7a). Real-time RT-PCR clearly showed that NAC significantly abolished the induction of TNFα mRNA expression and reduction of aggrecan in the AF tissues of the degenerative model. The mRNA expression of MMP-3 and COX-2 had a tendency to be decreased with oral administration of NAC in the degenerative model (Fig. 7b). To determine whether NAC neutralize the progression of oxidative stress in degenerative discs, we assessed the protein expression level of nitrotyrosine in AF tissues 1 month after surgery. Western blotting showed that the expression of nitrotyrosine was clearly reduced by oral administration of NAC (Fig. 7c). The densitometry confirmed these observations (Fig. 7d). Moreover, we found that the phosphorylation of p38 was obviously attenuated by treatment of NAC, but not JNK and ERK (Fig. 7e). Furthermore, H&E staining showed improvements in the reduction of NP size and the disorganization of the AF by oral administration of NAC (Fig. 7f). Finally, mid-sagittal T2-weighted MRI findings showed the maintenance of T2 high intensity in discs of the degenerative models with NAC (Fig. 7g). The ratio of the T2-weighted high-intensity area to IVD was significantly improved by the administration of NAC in the degenerative group (Fig. 7h).

## Discussion

This study demonstrated that excessive ROS, which are induced in IVD degeneration, have a catabolic effect on AF cells via MAPK signaling. Our study also showed that TNFα induced oxidative stress with increased intracellular ROS levels in AF cells through MAPK and NF-κB signaling, indicating that a positive feedback loop was formed between excessive ROS and catabolic factors, including TNFα, in IVD degeneration. The second major observation was that antioxidant NAC significantly abrogated the catabolic effect of excessive ROS both in vitro and in vivo. These findings lend strong support to the hypothesis that excessive ROS are critical mediators in the pathogenesis of degenerative disc conditions and are therapeutic targets for disc diseases.

In this study, we showed that nitrotyrosine, a product of tyrosine nitration mediated by ROS, was highly expressed in IVD in a rat degenerative model and in human degenerative samples. ROS also contribute to the onset and progression of osteoarthritis by inducing chondrocyte death and matrix degradation [37]. Tomiyama et al. [38] showed that compression on the cartilage induced the expression of nitrotyrosine. Furthermore, compared with healthy volunteers, plasma nitrotyrosine has been reported to be increased in osteoarthritis patients [39]. These observations indicated that nitrotyrosine was useful as an indirect oxidative stress marker in musculoskeletal degenerative diseases. An immuno-spin trapping technique which was recently developed by Khoo et al. [40] can be useful for direct detection of free radicals in future oxidative disc studies.

Many animal models of IVD degeneration have been reported; however, there are few ideal animal models which precisely mimic a pathological state of human IVD degeneration. A rat degenerative model induced by needle puncture has been used in many studies and has been shown to create morphological and biochemical features similar to many of those of degenerative discs in humans [41, 42]. Because it is clinically difficult to obtain a sufficient number of human healthy disc samples, the rat model is helpful for comparative study between degenerative and nondegenerative disc. However, the punctured model still has some limitations that should be taken into consideration, including the possibility of traumatic changes. We are assessing the expression level of nitrotyrosine in a rat-tail static compression-induced disc degeneration model to clarify the interaction between oxidative stress and mechanical loading in IVD degeneration.

Clinically, the characteristics of IVD degeneration such as ECM breakdown can be observed in both the AF and NP. The NP is known to have small chondrocyte-like cells and large vacuolated notochordal cells [6, 7]. Moreover, it has been reported that notochordal cells are rarely present after adolescence in humans [6]. However, it has not been determined whether the number of notochordal cells in the NP declines after birth because of their slow transformation or replacement by chondrocyte-like cells [43]. Which cells should be clinically targeted for the treatment of NP degeneration therefore remains unclear. On the other hand, although AF anatomically consists of inner and outer layers, the cells existing in both layers are known to originate from the mesenchyme and are homogeneous regardless of age and species. Therefore, it is reasonable to assume that alteration in the molecular phenotype of AF cells may be directly involved in IVD degeneration. From these viewpoints, in vitro analysis of AF cells was carried out to assess disc degeneration in this study.

**Fig. 8:**
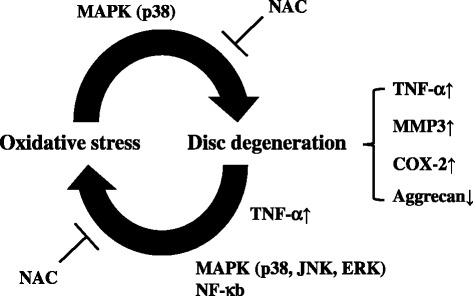
Schematic of relationship between oxidative stress and disc degeneration. Schematic showing positive catabolic feedback loop between excessive ROS and catabolic factors in AF cells. Excessive ROS induced expression of catabolic factors, which are upregulated in the degenerative state, and reduced cartilage ECM aggrecan via the signaling pathways of p38 in AF cells. Conversely, TNFα increased intracellular ROS levels in AF cells through p38, JNK, ERK, and p65. These pathways were neutralized by NAC. *COX* cyclooxygenase, *ERK* extracellular signal-regulated kinase, *JNK* c-Jun N-terminal kinase, *MAPK* mitogen-activated protein kinase, *MMP* matrix metalloprotease, *NAC* N-acetyl cysteine, *NF* nuclear factor, *TNF* tumor necrosis factor

IVD degeneration is characterized by increases in levels of the proinflammatory cytokines TNFα, IL-1α, IL-1β, IL-6, and IL-17, secreted by IVD cells [12, 13, 44, 45]. In particular, it is known that TNFα and IL-1β play pivotal roles in the progression of these degenerative changes [12, 13]. Cytokines have been shown to upregulate chemokines and various catabolic mediators, including ADAMTS-4, ADAMTS-5, MMP-1, MMP-2, MMP-3, MMP-13, and syndecan-4, and to suppress the expression of important ECM genes through NF-κB and MAPK signaling pathways in degenerative disc cells [14–17, 46, 47]. In a previous study, Yoshimura et al. [48] demonstrated that IL-1β induced cell death in rat primary chondrocytes and mouse chondrocytic ATDC5 cells via mitochondrial dysfunction in a ROS-dependent manner. Our flow cytometry analysis clearly showed that intracellular ROS levels were significantly induced by treatment with TNFα in AF cells. Furthermore, to evaluate oxidative stress conditions in TNFα-treated AF cells, a luciferase assay was carried out using OKD48 construct. Under oxidative stress conditions, Nrf2, a basic region leucine zipper transcription factor, is known to be stabilized at the posttranscriptional level [49]. OKD48 consists of an oxidative stress-inducible promoter, luciferase, and an Nrf2 fragment that contributes to stress-dependent stabilization [36]. This analysis clearly showed that oxidative stress was significantly induced by TNFα in AF cells. Vice versa, our results showed that the expression of catabolic factors, including TNFα, was induced by treatment with H_2_O_2_ or BSO in AF cells, which indicated that there was a reciprocal interaction between excessive ROS and TNFα in inflammatory AF cells (Fig. 8). We previously reported that ROS activated the ERK and p38 MAPK pathways but not the JNK pathway in ATDC5 cells [50]. On the other hand, the present study showed that JNK signaling was also activated by treatment of AF cells with ROS, suggesting that the downstream pathway of ROS is cell or tissue specific.

NAC is primarily a pharmaceutical drug for the management of acetaminophen overdose and is commonly used as a nutritional supplement. In addition, NAC is widely used for treatment of lung diseases such as chronic obstructive pulmonary disease [51, 52]. MRI and RT-PCR analysis in our in vivo study clearly showed that the oral administration of NAC prevented the progression of IVD degeneration. In analysis of molecular mechanisms, NAC treatment attenuated ROS-mediated or TNFα-mediated activation of MAPKs, including ERK1/2, p38, and JNK, and NF-κB signaling in AF cells in vitro. Previously, resveratrol in red wine and epigallocatechin 3-gallate in green tea were also reported to have an anti-inflammatory and anti-catabolic effect by modulating the signaling of p38, JNK, or NF-κB in IVD [53, 54]. Although several human clinical trials with anticytokine agents have investigated the alleviation of symptoms of back or radicular pain associated with IVD degeneration, there are no drugs that can directly prevent the progression of disc degeneration. Because the expression of p38 and p65 is ubiquitous, the inhibitors for targeting this signaling are often toxic to normal cells or tissue and have therefore been clinically unsuccessful [55]. Meanwhile, since NAC is commonly used as a supplement and has less toxic side effects, it may serve as a therapeutic option for IVD degeneration. When the human clinical daily dosage of NAC was converted to rats normalized with body surface area according to the US Food and Drug Administration’s guideline, they were almost equivalent doses. Experiments are in progress to determine the minimum effective concentration of NAC for the improvement of IVD degeneration. Recently, clinical use of another antioxidant, vitamin E, has been established for the treatment of nonalcoholic steatohepatitis in which oxidative stress was implicated [56]. Our results showed that vitamin E also attenuated the ROS-mediated or TNFα-mediated catabolic effect in the cultured AF cells, which indicated that vitamin E as well as NAC has the potential for preventing disc degeneration.

## Conclusions

Oxidative stress induced in IVD degeneration has a catabolic effect on AF cells via MAPK signaling. Elevated TNFα induces oxidative stress with increasing intracellular ROS levels through MAPK and NF-κB signaling, indicating that a positive feedback loop is formed between excessive ROS and TNFα in AF cells. Antioxidant NAC significantly abrogated the catabolic effect of excessive ROS in vitro and in vivo. These findings lead strong support for the hypothesis that NAC can be a therapeutic option for IVD degeneration.

## Additional files

Additional file 1:
**is Figure S1 showing molecular phenotype of the passaged rat AF cells.** Real-time RT-PCR analysis of the mRNA expression of type I collagen, type II collagen, and aggrecan in the second (P2) and fifth (P5) passaged AF cells. Data presented as mean ± SD of three independent experiments performed in triplicate (*n* = 3); **p* <0.05; ns, not significant. (PDF 873 kb) Additional file 2:
**is Figure S2 showing treatment of MAPK inhibitors to AF cells with ROS.** Real-time RT-PCR analysis of the expression of TNFα and MMP-3 in AF cells. MAPK signaling inhibitors, including p38 inhibitor (SB), JNK inhibitor (SP), and ERK inhibitor (PD), were treated to AF cells with H_2_O_2_ (upper) or BSO (lower). Data presented as mean ± SD of three independent experiments performed in triplicate (*n* = 3); **p* <0.05; ns, not significant. (PDF 987 kb)

## Abbreviations

ADAMTS: A disintegrin and metalloprotease with thrombospondin motifs
AF: Annulus fibrosus
AGE: Advanced glycation end product
ANOVA: Analysis of variance
BSO: Buthionine sulfoximine
COX-2: Cyclooxygenase-2
DMEM: Dulbecco’s modified Eagle’s medium
ECM: Extracellular matrix
ERK: Extracellular signal-regulated kinase
FBS: Fetal bovine serum
H_2_O_2_: Hydrogen peroxide
H&E: Hematoxylin and eosin
HPRT: Hypoxanthine phosphoribosyl transferase
HRP: Horseradish peroxidase
IL: Interleukin
IVD: Intervertebral disc
JNK: c-Jun N-terminal kinase
MAPK: Mitogen-activated protein kinase
MMP: Matrix metalloprotease
MRI: Magnetic resonance imaging
NAC: *N*-acetyl cysteine
NF: Nuclear factor
NO: Nitric oxide
NP: Nucleus pulposus
O^2−^: Superoxide anion
OH: Hydroxyl radical
ROS: Reactive oxygen species
SD: Standard deviation
TNFα: Tumor necrosis factor alpha
TPER: Tissue Protein Extraction Reagent
