# Supplementary figures and images for: Excessive reactive oxygen species are therapeutic targets for intervertebral disc degeneration
Source: Arthritis Res Ther. 2015 Nov 5;17:316. doi: 10.1186/s13075-015-0834-8 (PMC4635526; doi:10.1186/s13075-015-0834-8)

Supplementary Figure 1

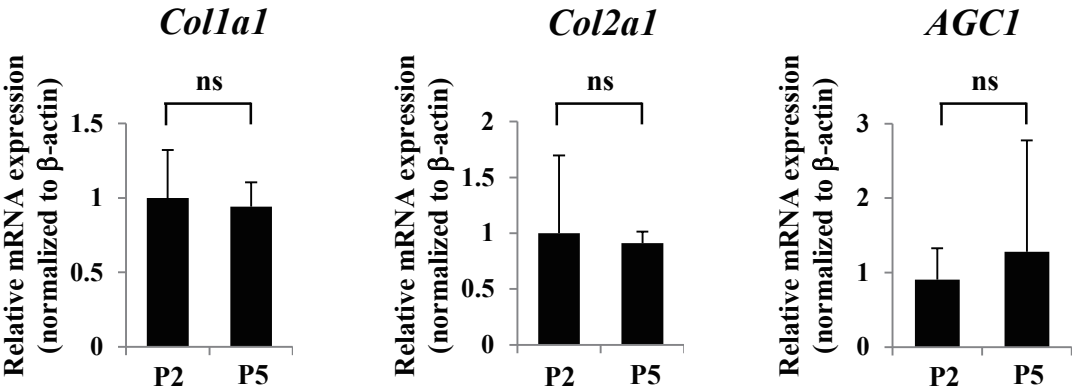

Supplement: Additional file 1: — is Figure S1 showing molecular phenotype of the passaged rat AF cells. Real-time RT-PCR analysis of the mRNA expression of type I collagen, type II collagen, and aggrecan in the second (P2) and fifth (P5) passaged AF cells. Data presented as mean ± SD of three independent experiments performed in triplicate (n = 3); *p <0.05; ns, not significant. (PDF 873 kb) [file 13075_2015_834_MOESM1_ESM.pdf]

Supplementary Figure 2

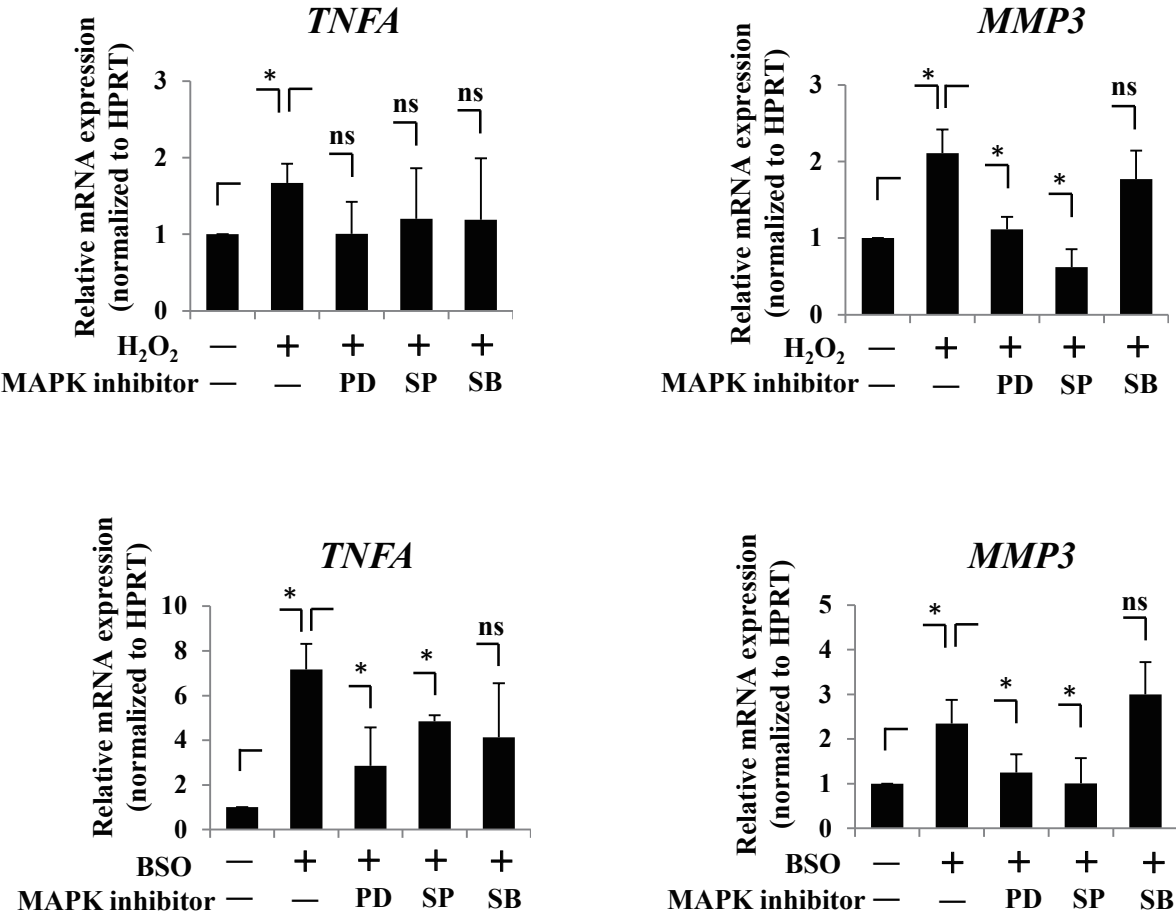

Supplement: Additional file 2: — is Figure S2 showing treatment of MAPK inhibitors to AF cells with ROS. Real-time RT-PCR analysis of the expression of TNFα and MMP-3 in AF cells. MAPK signaling inhibitors, including p38 inhibitor (SB), JNK inhibitor (SP), and ERK inhibitor (PD), were treated to AF cells with H2O2 (upper) or BSO (lower). Data presented as mean ± SD of three independent experiments performed in triplicate (n = 3); *p <0.05; ns, not significant. (PDF 987 kb) [file 13075_2015_834_MOESM2_ESM.pdf]
